# Outcomes and Complications of Pelvic Chondrosarcomas Treated Using Navigation Guidance and Multidisciplinary Approach: Is the Tumor Volume a Prognostic Factor?

**DOI:** 10.3390/jcm11237111

**Published:** 2022-11-30

**Authors:** Alberto Crimì, Odion T. Binitie, Filippo Crimì, G. Douglas Letson, David M. Joyce

**Affiliations:** 1 Department of Orthopedics and Orthopedic Oncology, University of Padova, 35128 Padua, Italy; 2 Sarcoma Department, H. Lee Moffitt Cancer Center and Research Institute, Tampa, FL 33612, USA; 3 Department of Orthopedic Surgery, University of South Florida College of Medicine, Tampa, FL 33612, USA; 4 Department of Medicine-DIMED, University of Padova, 35128 Padua, Italy; 5 Institute of Radiology, University Hospital of Padova, 35128 Padua, Italy

**Keywords:** pelvis chondrosarcoma, intraoperative navigation, hemipelvectomy, custom prosthesis

## Abstract

(1) Background: Pelvic Chondrosarcomas (CS) have a poor prognosis. The grade is the most important survival predictor; other factors are periacetabular location and Dedifferentiated CS subtype. The aim of the study is to investigate a series of CS of the pelvis, to analyze the prognostic factors that affect outcomes and to demonstrate how the use of intraoperative navigation can reduce the complications without worse outcomes. (2) Methods: Retrospective study on 35 patients (21 M, 14 F), median age at surgery 54 years (IQR 41–65), with pelvic CS, treated with hemipelvectomy under navigation guidance. (3) Results: 30 high-grade CS and 5 low-grade CS; mean follow-up 51.4 months. There was a positive linear correlation between the tumor volume and the presence of local recurrence at follow-up. The mean survival time of patients with larger chondrosarcoma volume was lower, but not significantly so. Lower MSTS score was associated with significantly lower survival time (*p* < 0.001). (4) Conclusion: in this series overall survival, LR and distant metastasis were comparable with recent literature, while complication rate was lower compared to similar series without the use of navigation. There was a correlation between tumor volume and local recurrence rate but not with the presence of metastasis at follow up.

## 1. Introduction

Chondrosarcomas (CS) are a heterogeneous group of malignant cartilage-matrix-producing tumors [1]. They are the most common bone sarcoma in adults, with 80% of CS cases diagnosed in patients older than 40 years of age [2]. Males and females are almost equally affected, with a slight predominance of males [3], and the overall incidence is 0.2/100,000/year [4]. The most common sites of primary bone sarcomas are in order of frequency: lower extremities (34%), pelvis (19% of the cases) and upper extremities (13%) [5]. CS shows a similar distribution, with the pelvis being the second-most common primary site of disease [2,6].

The presentation of pelvic CS is nonspecific, and it is often asymptomatic until it becomes large enough to have a mass effect on the pelvic organs [7]. The determination of grade is often challenging for the pathologist, given the heterogeneity of the tumor [8]. An experienced pathologist is pivotal in determining the correct grade of CS, despite reliability controversy [9].

The treatment for CS of the pelvis is surgical resection with wide margins [10], intralesional treatments of low-grade pelvic CS, is plagued with high rates of local recurrence [11,12].

Conventional CS does not respond to chemotherapy and radiation therapy [7,9,13]. The poor vascularity, the abundant extracellular matrix and the low percentage of dividing cells are thought to be the reasons why [14,15]. Chemotherapy can play a role in Mesenchymal and Dedifferentiated CS subtypes, which are more chemo-sensitive [16,17], while radiation therapy can have a palliative role in unresectable CS [14].

The prognosis of CS is influenced by different elements: grade, location, size, and stage [18], with poor prognosis in cases of locally advanced and metastatic high-grade chondrosarcomas [17]. In a recently published paper, the grade is defined as the only reliable prognostic factor for overall survival in CS [19]. Another paper highlighted local recurrences to be associated with the worst overall prognosis in high-grade conventional CS [20].

Pelvic CS has been shown in some studies to have worse outcome compared to extremity CS [4,21,22], while other studies have not demonstrated this association [23,24,25].

These doubts in the prognostic value of different factors are mainly due to the small number of series of pelvis CS described in the literature [11,26]. Specifically for pelvis CS, grade seems to be the most important survival predictor. Other relevant factors that have been described as associated with a lower survival rate are periacetabular location and Dedifferentiated CS subtype [27].

Computer-assisted navigation has been used in orthopedic surgery since the early 2000s [28], particularly in musculoskeletal oncology and pelvic sarcomas [29]. It manages to integrate preoperative imaging and intraoperative imaging, providing real-time high-definition three-dimensional feedback [30]. The images obtained have higher resolution than fluoroscopy [31]. Computer-assisted navigation was shown to improve precision and accuracy in bone tumor resections, leading to higher rate of margin-free resections [28,29,30]. On the counterpart, there is an increased procedure time of 15–47 min, which has been calculated to be approximately 6% of an extensive surgical procedure, such as a pelvic chondrosarcoma case [32].

The aim of the study was to investigate a series of CS of the pelvis, treated at a tertiary referral institution, to find factors influencing and associated with the outcome of CS patients and to demonstrate how a tailored soft tissue and bone preserving technique, made possible with the use of navigation, can reduce the complications without the burden of worse outcomes.

## 2. Materials and Methods

### 2.1. Design of the Study

A retrospective study was conducted, with the approval of Institutional Review Board, on patients with pelvic CS, treated with hemipelvectomy under navigation guidance from 2004 to 2021, at a tertiary level referral institution for diagnosis and treatment of sarcomas.

Inclusion criteria were localized CS of any grade, located in the pelvis, and treated with **a** hemipelvectomy under navigation guidance. Exclusion criteria were CS in extremity locations, CS involving only the sacrum, metastatic disease at presentation, incomplete medical records, and part of treatment performed at an outside hospital. For each patient, the following details were recorded: type and grade of chondrosarcoma, type of hemipelvectomy, type of reconstruction, margins status, volume of the tumor, presence of local recurrence, metastatic disease, post-operative complications, functional outcome (evaluated with MSTS score), oncological outcome and overall survival.

### 2.2. Surgical Technique

All the patients underwent staging at the time of diagnosis with radiographs, a CT of the thorax and an MRI with and without contrast of the pelvis. All patients also underwent a preoperative computed tomography (CT) scan with 1mm slice thickness and magnetic resonance imaging (MRI) with 3 mm slice thickness, as per protocol for navigation system (Stryker Orthomap 3D Navigation System: Stryker, Kalamazoo, MI, USA). Planes of resection were identified in the software and intraoperatively confirmed by correlations with C-arm 3D fluoroscopy unit or intraoperative CT [33]. The surgical approach was **a** utilitarian pelvic approach. The combined use of extended ilio-inguinal and ilio-femoral approach, in all the cases, was partly or completely used based on the type of resection [34]. Navigation was performed with the tracker placed with pins into an unaffected portion of the pelvis, and either a point-to-point registration with fiduciaries or automatic registration with preoperative CT was utilized. The accepted error was ≤ 1 mm. Osteotomies were performed with navigated tools (saw, osteotome, burr, ultrasound osteotome) [33]. Margins were divided into positive and negative, with the negative group subdivided into negative < 1 mm and negative > 1 mm from the resection margins [35]. The dimensions of the CSs were reported in volume (cm3), based on the pathology report of the three-dimensional measure. It was calculated using the ellipsoid formula (Volume = 4/3 × π × A × B × C, where A, B and C are the semiaxes of the measures obtained from the final pathology report)**.**

### 2.3. Statistical Analysis

Results are expressed as percentages, mean ± standard deviation (SD) or median and inter-quartile range (IQR), as appropriate. Continuous variables were tested for normal distribution with the Kolmogorov–Smirnov test. Comparisons were performed with parametric or nonparametric tests (Mann–Whitney), as appropriate. Pearson’s correlation test was used to study the correlation between variables.

A stepwise backward logistic regression analysis, using 0.05 and 0.10 as inclusion and exclusion criteria, respectively, was used to identify the best predictors of patients’ death, development of local recurrence and distant metastases at follow-up. The variables included in the initial model were the age of the patients, the CS grade, the tumor volume, the presence/absence of invaded margins, the presence/absence of wide margins and the MSTS score.

A receiver operating characteristic (ROC) curve was drawn using the de Long technique; the Youden index was used to identify the value corresponding to the best trade-off between sensitivity and specificity. Significance was set at *p* < 0.05. Kaplan–Meier survival curves were generated to compare overall survival between groups. SPSS (IBM Corp., Armonk, NY, USA) and MedCalc (MedCalc Software, Ostend, Belgium) were used.

## 3. Results

Thirty-eight patients were treated at a tertiary referral institution for a pelvic CS. One patient was excluded for involvement of the sacrum. Two patients were excluded because part of the treatment was performed at another institution. After the selection, 35 patients operated on for chondrosarcoma of the pelvis were reviewed: 21 were male and 14 female, and the median age at surgery was 54 years (IQR 41–65). The clinical data are reported in Table 1. The cohort consisted of 30 high grade CS (Grade 2, 3 or dedifferentiated) and 5 low-grade CS (Grade 1), defined by the final pathology report. The mean follow-up was 51.4 months (range: 2 months–15 years).

**Figure 1:**
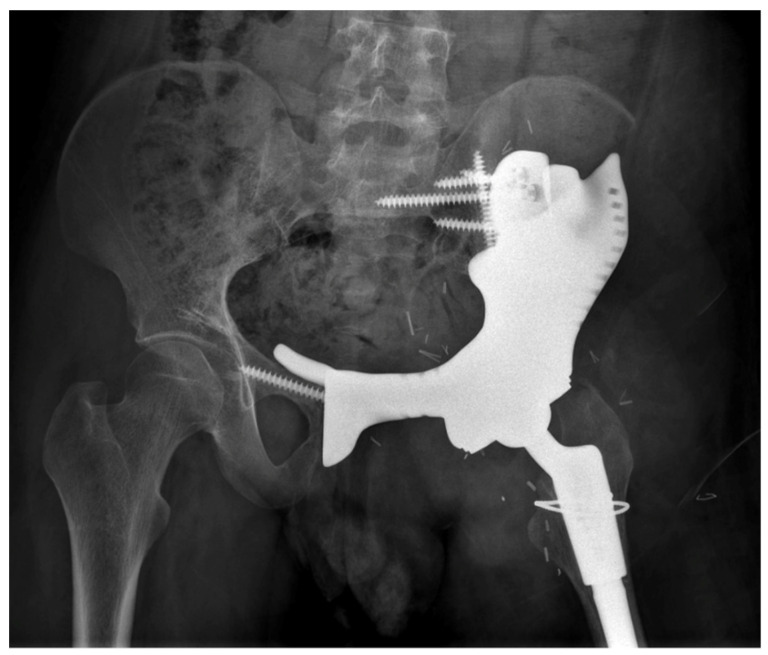
X-ray of a custom prosthetic reconstruction, for a type 2 resection of a grade 2 chondrosarcoma.

Twenty-six patients had a combined hemipelvectomy (Type I, II and III were considered). Six patients had a Type III resection, while three patients had a Type I resection. Meanwhile, 25 patients underwent a reconstruction following resection. The types of reconstructions were custom prosthesis in five cases (Figure 1), allograft in six cases, PARS (Peri-Acetabular Reconstruction System prosthesis: a fixed nonmobile modified saddle prosthesis) in seven cases, and a cage and cement construct in seven cases. All patients also had a hip joint replacement.

The margins were negative in 25 patients; 18 patients had a negative margin < 1 mm (close margin); and 7 patients had a negative margin > 1 mm (wide margin). Meanwhile, 10 patients had a positive margin. Two patients with positive margins had a pathologic fracture on CS. The tumor volume median was 683 cm^3^ (IQR 149–1151 cm^3^).

The overall survival rate was 60%. The local recurrence rate was 37% and the rate of distant metastasis was 31%. The oncologic outcome at the last follow-up was no evidence of disease (NED) for 18 patients, while 3 patients were alive with disease (AWD) and 14 patients were deceased. Of those: 10 patients died from the disease (DWD), while 4 patients died of other causes.

Complications were classified as either early (less than 30 days) or late (more than 30 days). Early complications were wound dehiscence, Deep Venous Thrombosis (DVT), prosthesis dislocations and nervous deficits. Late complications were deep infection and prosthesis mechanical failure. The wound complication rate was 28% in the reconstruction cases (considering both early and late complications).

Four patients (11.4%) had an external hemipelvectomy for local recurrence control, and out of these, one survived to the last follow-up. There were four DVTs and one pulmonary embolism (PE), for a total of five venous thrombotic events (VTE) (14%), all early complications. There were seven peripheral nerve complications (foot drop) (20%). The dislocation rate was 8% in acetabular reconstruction cases (two patients). The deep infection rate was 11.4% (four patients) in the overall sample and 16% among the reconstructed cases, with only one patient requiring hardware removal. the other three cases were managed with irrigation and debridement (I&D), substitution of mobile components and chronic oral suppression based on the microbiology susceptibilities. One patient had a mechanical failure of the prosthesis: a PARS that became loose 5 weeks after surgery. The patient was than treated with a Girdlestone procedure. The mean MSTS score of the sample was 20 (range 10–28).

From the statistical analysis, it was highlighted that there was a positive linear correlation between the tumor volume and the absence/presence of local recurrence at follow-up (r = 0.3432, *p* = 0.0435). The volume of the tumor at the time of surgery of patients that had local recurrence at follow-up was significantly higher than that of patients who did not experience local relapse of disease (median 1175 cc; IQR 711–1483 cc vs. median 280 cc, IQR 81–847; *p* = 0.0022). Notably, the volume of chondrosarcomas did not show a significant correlation with the presence of positive surgical margins (r = 0.3132; *p* = 0.0668), the presence of metastases at follow-up (r = −0.0662; *p* = 0.7056) or with post-operative complications (r = −0.1381, *p* = 0.4288).

Moreover, we investigated the prognosticators of patients’ death, development of local recurrence and distant metastases at follow-up, using stepwise backward logistic regression analyses by including all the most plausible predictors (the age of the patients, the CS grade, the tumor volume, the presence/absence of invaded margins, the presence/absence of wide margins and the MSTS score) in the initial model. 

For death and distant metastases development, no variables were retained in the model.

For local recurrences, collectively, the presence of invaded margins (coefficient = 2.61544; *p* = 0.0083) and the tumor volume (coefficient = 0.0011; *p* = 0.0903), the latter with borderline level of significance, predicted 77.1% of the variance of the outcome variable (Chi-squared = 15.018; *p* = 0.0005, Cox & Snell R^2^ = 0.3489).

**Figure 2:**
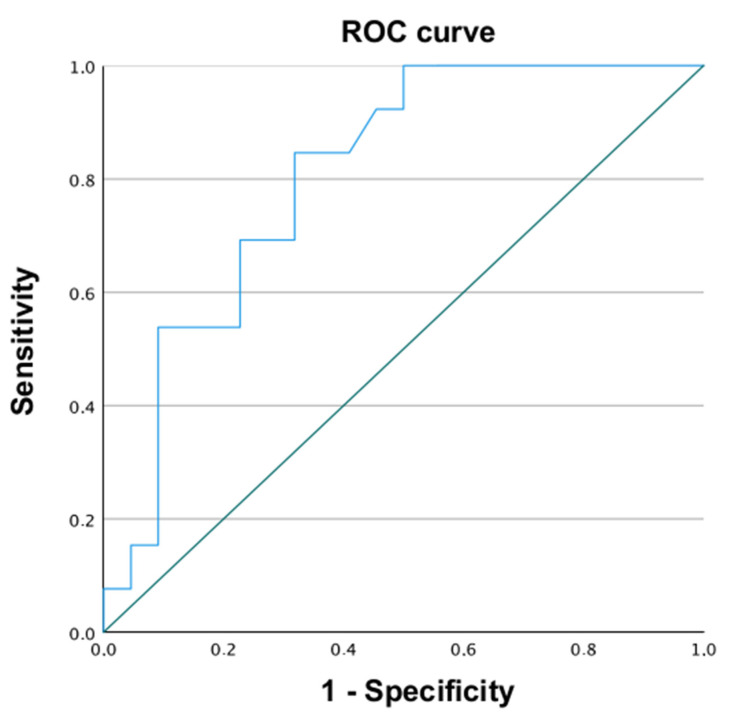
The area under the ROC (receiver operating characteristic) curve (AUC) was significantly higher than the area under the identity line confirming accuracy of volume predicting local recurrence.

A ROC curve was drawn to evaluate the association of chondrosarcoma volume at surgery and the prediction of local recurrences. The area under the ROC curve (AUC) was significantly higher than that under the identity line (0.813, 95% Confidence Interval [CI]: 0.645–0.924; *p* < 0.0001), confirming an association with the volume (Figure 2). The Youden index analysis identified as best cut off the value of 320 cc, with a sensitivity of 100% and a specificity of 54%, in predicting a local recurrence.

**Figure 3:**
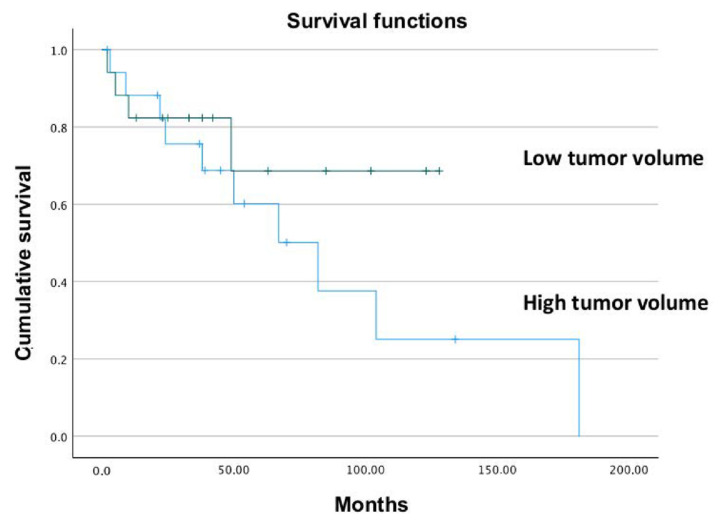
The mean survival time of patients with higher chondrosarcoma volume was lower but not significant.

We then analyzed the correlation between tumor volume and overall survival, comparing the low-volume group (tumor volume < 320 cc) and the high-volume tumor group (tumor volume > 320 cc) using Kaplan–Meier survival analysis (Figure 3). The mean overall survival of patients with high-volume chondrosarcomas was lower but not significantly different from low-volume tumors (mean overall survival 90 vs. 108 months, hazard ratio [HR]: 2.1, 95% CI: 0.6–6.8; *p* = 0.2230).

**Figure 4:**
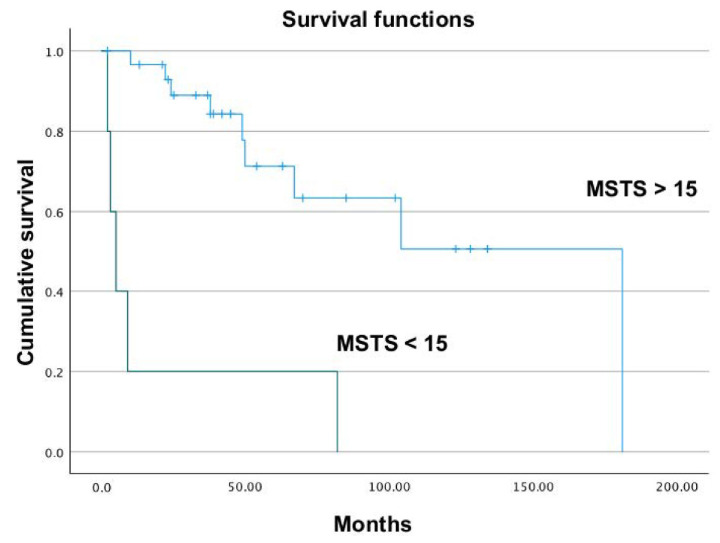
Lower MSTS (Musculo-Skeletal Tumor Society) score showed a significantly lower survival time.

The difference in overall survival between MSTS > 15 and MSTS < 15 group was compared (Figure 4), showing that the group with a lower MSTS score showed also a significantly shorter survival time.

Overall survival between patients with high-grade CS and low-grade CS and between patients with acetabular location of the CS and patients with CS that did not involve the acetabulum were compared. The low-grade CS group showed a longer survival time compared to the high-grade CS group, but this did not reach statistical significance (*p* = 0.0890), probably due to the small size of the population and the low number of CS grade I in the sample (5/35 patients). The overall survival between CS involving the acetabulum (type 2) and those that did not (types 1 and 3) did not show statistically significant differences in the survival time between the two groups (*p* = 0.5300).

## 4. Discussion

Chondrosarcoma of the pelvis has a worse outcome compared to extremity chondrosarcoma. In general, high-grade chondrosarcomas have a lower survival and higher local recurrence and metastasis rates [27].

The main treatment for CS of the pelvis is surgical resection with wide margins [10], since conventional CS does not respond to chemotherapy and radiation [7,9,13]. Wide resection is mandatory for Grade I extra-compartmental, grade II, grade III and dedifferentiated CS of the pelvis. A wide resection is also suggested for intra-compartmental CS grade I of the pelvis for the high risk of local recurrence (3–41%) with intralesional curettage [11].

The prognosis of CS is influenced by different elements: grade, location, size, and metastatic presentation [18]. The percent of CS Grade I in our sample was 14%, comparable to other recent works: 26.9% in Mavrogenis et al. [27] and 14% in Fujiwara et al. [36]. The evaluation of histology grade of a CS is a debated topic, varying among the evaluations from different pathologists [14,37,38]. There are some subtle distinctions between enchondroma and Grade I CS. Additionally, Grade I and Grade II chondrosarcoma are not always easily distinguishable. In some cases, molecular markers are necessary to further define the correct histological grade [39], as well as imaging characteristics. As in previous studies on CS of the pelvis, we based the grading on the final pathology report and when doubt was present following combined imaging and histological interpretation [14,40].

In this series, overall survival, local recurrence and metastatic rate were comparable with recent literature. In a SEER (Surveillance, Epidemiology and End Results) database-based analysis of almost the same period of our study, the overall survival was 54% at 10 years for conventional CS and 34% at 5 years for dedifferentiated CS [41]. The analysis of prognostic factors in a recent series, treated with modern techniques, can update the scientific literature and refine some prognostic factors based on historical series and data [11,27,34].

This study has some limitations. The sample is small, and follow-up was in some cases not long enough to evaluate statistical significative correlations of some elements of the study, despite a trend being noticed. There is the lack of a population treated without navigation to compare. Surgeons involved in treatment also reviewed the data, so some assessment bias could be possible.

Unlike a recent paper [27], we did not find a statistically significant difference in overall survival and rate of local recurrence and metastatic rate in patients with acetabular localization of the CS and patients without acetabular involvement by the CS. Moreover, discordantly with the previously cited study [27], we did not find any correlation between the dimension of the CS and the presence of metastasis at follow-up, while there was a statistically significant correlation between the volume of the CS and the local recurrence rate. With the ROC curve, we found that the cut-off volume of 320 cm^3^ predicts a local recurrency, with a sensitivity of 100% and a specificity of 54%. The interpretation of this result was that the tumor biological behavior is affecting the size of the tumor itself, and the bigger the tumor, the more aggressively it will behave. Consequently, it has a higher likelihood of local recurrence.

To better evaluate the real dimension of the CS, the linear measure was not felt to be representative, given that tumors did not present as perfect spheres. Instead, we felt the measured volume of the tumor, based on the pathology result, would have a more precise characterization of the real size. It can be argued that the volumetric measure is approximative, giving the volume measured with the three maximal dimensions written in the pathology report. These measures were always confirmed on preoperative imaging measures, and although an approximation, it gave a better perception of the dimension of CS and of its soft tissue component, and possibly a better understanding of the true behavior of the sarcoma.

It is interesting to notice that the LR rate was found to be a predictor of disease-specific survival in another work [42]. However, when the cut-off volume of 320 cm^3^ to subdivide the sample in two groups, high volume (>320 cm^3^) and low volume (<320 cm^3^) was used, it was noted a trend of lower survival in high volume group, but this difference was not statistically significant in the Kaplan–Meyer curve analysis (mean survival time 90 vs. 108 months, hazard ratio [HR]: 2.1, 95% CI: 0.6–6.8; *p* = 0.2230). The logistic regression statistical analysis showed that, among the variables considered, the invaded margins and the tumor volume were predictors of local recurrence, the volume at borderline significance. This trend could probably be confirmed with a larger sample of patients.

One of the greatest difficulties in the treatment of CS of the pelvis is their proximity, mainly due to size, to important vascular and nervous structures. Surgical margins have a pivotal role in the rate of local recurrence in previous papers [11,23,25,43,44]. Positive surgical margins and local recurrence rate are higher in CS of the pelvis compared to extremities CS, and the rate of contaminated margins reaches the 53% [4,23,24,25,43,44]. The use of navigation has shown better outcomes in local control and a reduced rate of contaminated margins [45]. In other recent series treated with navigation, the local recurrence rate (observed in the soft tissues) was 35% [46]. The issue of margins around the soft tissues is not solved by the use of computer navigation, which is better targeted to the management of the bone margins [36]. We tried to reduce this limitation coupling the CT protocol for navigation to an MRI of the pelvis with navigation protocol.

The analysis of margins status showed that when margins were negative, there was no difference in LR or metastasis based on the size of the margin >1 mm or <1 mm from the tumor. The relevant factor seems to be to obtain negative margins more than an extremely “wide” margin, which is always difficult to obtain in pelvic resections when very relevant structures such as the sciatic nerve, iliac vessels and other important structures are to be spared. In 10 cases, there was a positive margin, despite an intraoperative frozen section of the marrow margin that was negative.

This may also support the concept that the more difficult aspect of chondrosarcoma resection is the soft-tissue component management and the ability to obtain a negative margin, as well the difficulty in obtaining accessibility for the osteotomies of certain bone locations. Another possible hypothesis is that the larger the tumor, the more likely it is that pathology will miss a violation of tumor in the soft tissues, while it is easier for pathology to localize the bone margins as the bone cuts represent a smaller surface area to evaluate.

The MSTS score when <15 was correlated with worse survival. The group with MSTS < 15 showed a mean survival time of 20 months, compared to the 121 months of the group with MSTS > 15 (HR: 107, 95% CI: 13–883; *p* < 0.001). MSTS was thus confirmed to be a good general function index and, in our series, correlated with the outcome of patients with CS of the pelvis. Patients who performed worse during the post-operative follow-up were also the patients with the worst survival.

Analyzing the complications, lower dislocation rates were found compared to a similar work [38] in the literature: 8% vs. 32%. Infection rates were also lower compared to similar works: 16% vs. 38%. When including superficial wound dehiscence, our rate reached 28%; however, these were managed by local wound care.

This low infection rate may be for several reasons, the first of which is that antibiotics were provided for a minimum of 3 weeks after surgery, with both coverage for Gram + and Gram-, as tailored by Orthopedics in conjunction with the Infectious Disease team. Plastic surgery was also involved in every case, evaluating the patient before surgery, and providing a muscular flap envelope around the reconstructions and under the areas of the skin flaps that traditionally have healing issues. Surgical oncologists were involved in the dissection of main vessels and pelvic organs, mainly when large soft-tissue components were highlighted by the preoperative imaging. The use of navigation to minimize dissection and to preserve bone may help with both the dislocation rate, as well as infection/wound breakdown rates. Additional research in these areas is needed.

## 5. Conclusions

Mortality and events-free survival results were in line with the literature on pelvis CS. Tumor volume at surgery was found to be a good predictor of local recurrence, and functional outcome was found to be strictly correlated with survival. There were less mechanical failures and infections compared to other series in the literature. The multidisciplinary approach, the use of soft-tissue and bone-preserving techniques, made possible by navigation assisted surgery, led to a comparable survival rate and lower complication rate.

Pelvis CS is a rare condition, which is difficult to treat due to the burden of complications and overall poor prognosis if compared to other sites. Further multicentric studies, with selected cases and advanced techniques, can improve the scientific literature, providing a better understanding of the behavior of the disease, and thus offering better treatment for patients.

## Figures and Tables

**Table 1:** Demographic, oncological and complications data of the population.

Features	Classification	Value
Sex	M (*n*, %) F (*n*, %)	21, 60% 14, 40%
Age	Median, (IQR)	54 years (41–65)
Grade	High (*n*, %) Low (*n*, %)	30, 86% 5, 14%
Oncologic outcome	NED AWD DWD D other D	18 patients 3 patients 10 patients 4 patients
MSTS score	(Median, Range)	20 (10–28)
Volume of the tumor	(Median, IQR)	683 cc (149–1151)
Local Recurrence Rate	%	37%
Metastasis rate	%	31%
Infection rate	%	16%
Dislocation rate	%	8%

MSTS (Musculo-Skeletal Tumor Society), NED (No Evidence of Disease), AWD (Alive With Disease), DWD (Died With Disease), D other D (Died of other Disease), IQR (Interquartile Range).

## Data Availability

The data presented in this study are available on request from the corresponding author. The data are not publicly available due to privacy reasons.

## References

[1] The WHO Classification of Tumours Editorial Board (2020). WHO Classification of Tumours Soft Tissue and Bone Tumours.

[2] Gatta G., Capocaccia R., Botta L., Mallone S., De Angelis R., Ardanaz E., Comber H., Dimitrova N., Leinonen M.K., Siesling S. (2017). Burden and centralised treatment in Europe of rare tumours: Results of RARECAREnet—A population-based study. Lancet Oncol..

[3] Weinschenk R.C., Wang W.-L., Lewis V.O. (2021). Chondrosarcoma. J. Am. Acad. Orthop. Surg..

[4] Giuffrida A.Y., Burgueno J.E., Koniaris L.G., Gutierrez J.C., Duncan R., Scully S.P. (2009). Chondrosarcoma in the United States (1973 to 2003): An Analysis of 2890 Cases from the SEER Database. J. Bone Jt. Surg..

[5] Lawrenz J.M., Curtis G.L., Styron J.F., George J., Anderson P.M., Zahler S., Shepard D.R., Rubin B.P., Nystrom L.M., Mesko N.W. (2018). Adult Primary Bone Sarcoma and Time to Treatment Initiation: An Analysis of the National Cancer Database. Sarcoma.

[6] Amadeo B., Penel N., Coindre J.-M., Ray-Coquard I., Ligier K., Delafosse P., Bouvier A.-M., Plouvier S., Gallet J., Lacourt A. (2020). Incidence and time trends of sarcoma (2000–2013): Results from the French network of cancer registries (FRANCIM). BMC Cancer.

[7] Guo W., Li D., Tang X., Ji T. (2010). Surgical treatment of pelvic chondrosarcoma involving periacetabulum. J. Surg. Oncol..

[8] Unni K.K. (2001). Cartilaginous lesions of bone. J. Orthop. Sci..

[9] Angelini A., Guerra G., Mavrogenis A.F., Pala E., Picci P., Ruggieri P. (2012). Clinical outcome of central conventional chondrosarcoma. J. Surg. Oncol..

[10] Strauss S.J., Frezza A.M., Abecassis N., Bajpai J., Bauer S., Biagini R., Bielack S., Blay J.Y., Bolle S., Bonvalot S. (2021). Bone sarcomas: ESMO–EURACAN–GENTURIS–ERN PaedCan Clinical Practice Guideline for diagnosis, treatment and follow-up. Ann. Oncol..

[11] Donati D., Ghoneimy AEl Bertoni F., Di Bella C., Mercuri M. (2005). Surgical treatment and outcome of conventional pelvic chondrosarcoma. J. Bone Jt. Surg. Br..

[12] Normand A.N., Cannon C.P., Lewis V.O., Lin P.P., Yasko A.W. (2007). Curettage of Biopsy-diagnosed Grade 1 Periacetabular Chondrosarcoma. Clin. Orthop. Relat. Res..

[13] Biermann J.S., Chow W., Reed D., Lucas D., Adkins D.R., Agulnik M., Benjamin R.S., Brigman B., Budd G.T., Curry W.T. (2017). NCCN Guidelines Insights: Bone Cancer, Version 2.2017. J. Natl. Compr. Cancer Netw..

[14] Gelderblom H., Hogendoorn P.C., Dijkstra S.D., Van Rijswijk C.S., Krol A.D., Taminiau A.H., Bovee J.V. (2008). The Clinical Approach Towards Chondrosarcoma. Oncologist.

[15] Geirnaerdt M.J., Hermans J., Bloem J.L., Kroon H.M., Pope T.L., Taminiau A.H., Hogendoorn P.C. (1997). Usefulness of radiography in differentiating enchondroma from central grade 1 chondrosarcoma. Am. J. Roentgenol..

[16] Frezza A.M., Cesari M., Baumhoer D., Biau D., Bielack S., Campanacci D.A., Casanova J., Esler C., Ferrari S., Funovics P.T. (2015). Mesenchymal chondrosarcoma: Prognostic factors and outcome in 113 patients. A European Musculoskeletal Oncology Society study. Eur. J. Cancer.

[17] Italiano A., Mir O., Cioffi A., Palmerini E., Piperno-Neumann S., Perrin C., Chaigneau L., Penel N., Duffaud F., Kurtz J. (2013). Advanced chondrosarcomas: Role of chemotherapy and survival. Ann. Oncol..

[18] Fromm J., Klein A., Baur-Melnyk A., Knösel T., Lindner L., Birkenmaier C., Roeder F., Jansson V., Dürr H.R. (2018). Survival and prognostic factors in conventional central chondrosarcoma. BMC Cancer.

[19] Laitinen M.K., Evans S., Stevenson J., Sumathi V., Kask G., Jeys L.M., Parry M.C. (2021). Clinical differences between central and peripheral chondrosarcomas. Bone Jt. J..

[20] Thorkildsen J., Taksdal I., Bjerkehagen B., Norum O., Myklebust T.A., Zaikova O. (2020). Risk stratification for central conventional chondrosarcoma of bone: A novel system predicting risk of metastasis and death in the Cancer Registry of Norway cohort. J. Surg. Oncol..

[21] Lee F.Y., Mankin H.J., Fondren G., Gebhardt M.C., Springfield D.S., Rosenberg A.E., Jennings L.C. (1999). Chondrosarcoma of Bone. J. Bone Jt. Surg..

[22] Björnsson J., McLeod R.A., Unni K.K., Ilstrup D.M., Pritchard D.J. (1998). Primary chondrosarcoma of long bones and limb girdles. Cancer.

[23] Sheth D.S., Yasko A.W., Johnson M.E., Ayala A.G., Murray J.A., Romsdahl M.M. (1996). Chondrosarcoma of the pelvis: Prognostic factors for 67 patients treated with definitive surgery. Cancer.

[24] Rizzo M., Ghert M.A., Harrelson J.M., Scully S.P. (2001). Chondrosarcoma of Bone. Clin. Orthop. Relat. Res..

[25] Kreicbergs A., Boquist L., Borssén B., Larsson S.-E. (1982). Prognostic factors in chondrosarcoma. A comparative study of cellular DNA content and clinicopathologic features. Cancer.

[26] Fiorenza F., Abudu A., Grimer R.J., Carter S.R., Tillman R.M., Ayoub K., Mangham D.C., Davies A.M. (2002). Risk factors for survival and local control in chondrosarcoma of bone. J. Bone Jt. Surg. Br..

[27] Mavrogenis A.F., Angelini A., Drago G., Merlino B., Ruggieri P. (2013). Survival analysis of patients with chondrosarcomas of the pelvis. J. Surg. Oncol..

[28] Wong K.C., Kumta S.M. (2013). Computer-assisted tumor surgery in malignant bone tumors. Clin. Orthop. Relat. Res..

[29] Cheong D., Letson G.D. (2011). Computer-assisted navigation and musculoskeletal sarcoma surgery. Cancer Control.

[30] Gerbers J.G., Stevens M., Ploegmakers J.J., Bulstra S.K., Jutte P.C. (2014). Computer-assisted surgery in orthopedic oncology: Technique, indications, and a descriptive study of 130 cases. Acta Orthop..

[31] Ieguchi M., Hoshi M., Takada J., Hidaka N., Nakamura H. (2012). Navigation-assisted surgery for bone and soft tissue tumors with bony extension. Clin. Orthop. Relat. Res..

[32] Young P.S., Bell S.W., Mahendra A. (2015). The evolving role of computer-assisted navigation in musculoskeletal oncology. Bone Jt. J..

[33] Joyce D.M. (2021). Navigation in Pelvic Surgery. Surgery of Pelvic Bone Tumors.

[34] Angelini A., Crimì A., Pala E., Ruggieri P. (2021). Surgical Approaches in Pelvic Bone Tumors. Surgery of Pelvic Bone Tumor.

[35] Bertrand T.E., Cruz A., Binitie O., Cheong D., Letson D.G. (2016). Do Surgical Margins Affect Local Recurrence and Survival in Extremity, Nonmetastatic, High-grade Osteosarcoma?. Clin. Orthop. Relat. Res..

[36] Fujiwara T., Kaneuchi Y., Stevenson J., Parry M., Kurisunkal V., Clark R., Tsuda Y., Laitinen M., Grimer R., Jeys L. (2021). Navigation-assisted pelvic resections and reconstructions for periacetabular chondrosarcomas. Eur. J. Surg. Oncol..

[37] Skeletal Lesions Interobserver Correlation among Expert Diagnosticians (SLICED) Study Group (2007). Reliability of histopathologic and radiologic grading of cartilaginous neoplasms in long bones. J. Bone Jt. Surg. Am..

[38] Eefting D., Schrage Y.M., Geirnaerdt M.J., Le Cessie S., Taminiau A.H., Bovée J.V., Hogendoorn P.C. (2009). Assessment of interobserver variability and histologic parameters to improve reliability in classification and grading of central cartilaginous tumors. Am. J. Surg. Pathol..

[39] Bovée J.V., Cleton-Jansen A.M., Taminiau A.H., Hogendoorn P.C. (2005). Emerging pathways in the development of chondrosarcoma of bone and implications for targeted treatment. Lancet Oncol..

[40] Rosenthal D.I., Schiller A.L., Mankin H.J. (1984). Chondrosarcoma: Correlation of radiological and histological grade. Radiology.

[41] Brown J.M., Rakoczy K., Hart J., Jones K.B., Groundland J.S. (2022). Presenting features and overall survival of chondrosarcoma of the pelvis. Cancer Treat. Res. Commun..

[42] Kurisunkal V., Laitinen M.K., Kaneuchi Y., Kapanci B., Stevenson J., Parry M.C., Reito A., Fujiwara T., Jeys L.M. (2021). Is 2 mm a wide margin in high-grade conventional chondrosarcomas of the pelvis?. Bone Jt. J..

[43] Kyoo-Ho S., Rougraff B.T., Simon M.A. (1994). Oncologic outcome of primary bone sarcoma of the pelvis. Clin Orthop..

[44] Ozaki T., Lindner N., Hillmann A., Rödl R., Blasius S., Winkelmann W. (1996). Influence of intralesional surgery on treatment outcome of chondrosarcoma. Cancer Interdiscip. Int. J. Am. Cancer Soc..

[45] Jeys L., Matharu G.S., Nandra R.S., Grimer R.J. (2013). Can computer navigation-assisted surgery reduce the risk of an intralesional margin and reduce the rate of local recurrence in patients with a tumour of the pelvis or sacrum?. Bone Jt. J..

[46] Nandra R., Matharu G., Stevenson J., Parry M., Grimer R., Jeys L. (2019). Long-term outcomes after an initial experience of computer-navigated resection of primary pelvic and sacral bone tumours: Soft-tissue margins must be adequate to reduce local recurrences. Bone Jt. J..

